# The implication of holocytochrome c synthase mutation in Korean familial hypoplastic amelogenesis imperfecta

**DOI:** 10.1007/s00784-022-04413-0

**Published:** 2022-03-03

**Authors:** Hyejin Choi, Kwanghwan Lee, Donghyo Kim, Sanguk Kim, Jae Hoon Lee

**Affiliations:** 1 Department of Prosthodontics, College of Dentistry at Yonsei University, 50-1 Yonsei-ro, Seodaemoon-gu, Seoul, 120-752 Republic of Korea; 2 Department of Life Sciences, Pohang University of Science and Technology, Pohang, 790-784 Republic of Korea

**Keywords:** Amelogenesis imperfecta, Whole-exome sequencing, *HCCS*, Bioinformatics analysis, Korean population

## Abstract

**Objectives:**

This study aimed to comprehensively characterise genetic variants of amelogenesis imperfecta in a single Korean family through whole-exome sequencing and bioinformatics analysis.

**Material and methods:**

Thirty-one individuals of a Korean family, 9 of whom were affected and 22 unaffected by amelogenesis imperfecta, were enrolled. Whole-exome sequencing was performed on 12 saliva samples, including samples from 8 affected and 4 unaffected individuals. The possible candidate genes associated with the disease were screened by segregation analysis and variant filtering. In silico mutation impact analysis was then performed on the filtered variants based on sequence conservation and protein structure.

**Results:**

Whole-exome sequencing data revealed an X-linked dominant, heterozygous genomic missense mutation in the mitochondrial gene holocytochrome c synthase (*HCCS*). We also found that *HCCS* is potentially related to the role of mitochondria in amelogenesis. The *HCCS* variant was expected to be deleterious in both evolution-based and large population-based analyses. Further, the variant was predicted to have a negative effect on catalytic function of *HCCS* by in silico analysis of protein structure. In addition, *HCCS* had significant association with amelogenesis in literature mining analysis.

**Conclusions:**

These findings suggest new evidence for the relationship between amelogenesis and mitochondria function, which could be implicated in the pathogenesis of amelogenesis imperfecta.

**Clinical relevance:**

The discovery of HCCS mutations and a deeper understanding of the pathogenesis of amelogenesis imperfecta could lead to finding solutions for the fundamental treatment of this disease. Furthermore, it enables dental practitioners to establish predictable prosthetic treatment plans at an early stage by early detection of amelogenesis imperfecta through personalised medicine.

**Supplementary Information:**

The online version contains supplementary material available at 10.1007/s00784-022-04413-0.

## Introduction

Amelogenesis imperfecta (AI) is an encompassing term used to describe the presence of numerous abnormal forms of enamel. Many cases of AI are inherited via autosomal dominant, autosomal recessive, and X-linked mechanisms [1]. The incidence of AI varies from 1:714 to 1:14,000, depending on the region [2]. Patients with AI may have dental sensitivity, loss of occlusal vertical dimension, and difficulty maintaining oral hygiene [3]. These problems lead to disability of the masticatory function and lead to poor quality of life [4]. Mastication is important not only for food intake but also for general systemic and physical functions [5]. In addition, AI can also cause aesthetic problems and low self-esteem in individuals suffering from the disease. Affected patients show reduced chances of social interaction and reduced self-consciousness because of the characteristic aspects of their facial appearance [4].

AI can be classified into four different phenotypes. Based on these phenotypes (hypoplastic, hypomaturation, hypocalcified, and hypomaturation-hypoplastic), AI was subdivided into 15 subtypes and subsequently classified by the defining characteristics of the inherited trait. Among these phenotypes, the hypoplastic type does not achieve the normal thickness of enamel, but it is distinguished from dentin by showing normal enamel density in radiograph [6]. However, different families with the disease have very distinct traits, and it is difficult to clearly perform according to subgroup classification. In addition, although the AI phenotypes are largely classified into four types, the method of dividing the detailed phenotypes is not yet clear, and these are classified in various ways [1]. This is likely because different families may have distinct characteristics, with possibly different aetiologies. Depending on the phenotype of AI, in which part of the mechanism for generating enamel damage is caused can be explained differently. Therefore, we conducted research on a single family to avoid the inclusion of other phenotypes of AI with different aetiologies. This would make it possible to clearly investigate the cause.

AI affects the tooth enamel structure and appearance. Most of the amelogenesis processes leading to the formation of the enamel are known to be affected by associated genetic variations [7, 8]. For example, mutations in the *AMELX* [9], *ENAM* [10], *MMP20* [11], *KLK-4* [12], *FAM83H* [13], *WDR72* [14], *C4ORF26* [15], *SLC24A4* [16], *LAMB3* [17], and *ITGB6* [16] genes have been found and known to cause AI (non-syndromic form). *AMELX* and *ENAM* encode extracellular matrix proteins of the developing tooth enamel, and *KLK-4* and *MMP20* encode proteases that help degrade organic matter from the enamel matrix during the maturation stage of amelogenesis [9–12, 16]. *SLC24A4* encodes a calcium transporter that mediates calcium transport to developing enamel during tooth development [16].

Amelogenesis can be divided into four stages: presecretory, secretory, transition, and maturation. In the presecretory phase, ameloblasts begin to secrete enamel matrix proteins (EMP), and in the transition phase, the secretion of these proteins decreases, and ameloblasts are reconstituted [18]. In the maturation stage, the width and thickness of the enamel crystals increase [16]. Finally, the matrix is transformed into a mature enamel with little protein [19]. For the amelogenesis process to work normally, ameloblasts must keep contact with the secreted extracellular matrix during the secretion, transition, and maturation stages. Many of the genes reported to cause AI are either related to cell-cell matrix adhesion proteins or EMP. Depending on the cause, genetically occurring mutations produce a variety of phenotypes [20].

Genetic studies of AI have been conducted since the early 2000s. Candidate gene approaches were mainly focused on protein synthesis genes involved in the amelogenesis process. Sequence changes of the candidate genes in AI patients were identified, and the effects of each sequence variation on protein expression and structure were deduced [21, 22]. Due to the increased understanding of the amelogenesis process, new candidate genes, such as *SLC4A4* (a sodium bicarbonate cotransporter), have been discovered [23]. This candidate gene approach attempted to identify the main functions of these genes through animal experiments using knockout mice having null mutations and to confirm the central role of proteins, such as amelogenin [24] and enamelysin [11]. However, because of the severe heterogeneity between the groups of families, the candidate gene approach could not account for AI pathogenesis in which these candidate gene mutations were not identified. In such cases, AI-causing mutations may lie on different genes or loci that were not considered in previous studies. Therefore, additional studies are required to expand the repository of AI-causing genes [25].

With the development of genome research and DNA chip technology, genomic information can be obtained economically. The genome-wide association study (GWAS) in 2002 was the first attempt to explore genetic factors for diseases collectively. AI-related GWAS was conducted using 14 enamel hypoplasia (resembling human AI) samples from Italian greyhounds and 45 individuals in a healthy control group. This study confirmed a specific functional mutation in *ENAM* and revealed its recessive inheritance pattern [26].

The lower cost of next-generation sequencing has accelerated the discovery of mutations that cause AI [16]. These findings contributed to a better understanding of amelogenesis pathogenesis, including cell-cell adhesion, cell matrix adhesion, intercellular transport, signalling regulation, and the enamel mineralisation process during amelogenesis. The regulation of genes involved in these processes plays an important role in enamel formation.

In this study, we discovered a new variant in *HCCS* associated with AI in Koreans via whole-exome sequencing (WES). In silico mutation analysis based on sequence conservation and protein structure revealed deleterious effect of variant on *HCCS* function, affecting the mitochondria function. Further, literature mining analysis showed the potential association between this variant and the pathogenesis of amelogenesis. The new variant in *HCCS*, which is a mitochondrial protein, will help understand the relationship between the process of AI and dysfunction of mitochondria.

## Materials and methods

### Subjects

A family comprising 31 members across four generations was enrolled in this study. Of the 31 members, 9 were affected, and 22 were unaffected. Written consents were obtained for using the clinical medical information from 12 individuals of the family, including 8 subjects affected by AI. Since four of the participants were minors, written consent was obtained from their guardians. Saliva samples were collected to extract DNA. We analysed the family members affected by AI who gave their informed consent; these participants had no specific disease or clinical history besides amelogenesis imperfecta. This clinical research was approved by the Institutional Review Board of Yonsei University College of Dentistry (Yonsei IRB No. 2-2018-0055, Approved on 28 January 2019). This clinical study was conducted in accordance with the Helsinki Declaration.

### Clinical/Radiographic assessment and measurements

The diagnosis of AI was based on clinical and radiological evidence.

### Sample collection

From each consenting individual, 2 ml of saliva was collected using the Oragene DNA Self-Collection Kit (DNA Genotek Inc., Ottawa, Canada). A preservative solution in the tube was mixed with the saliva. Samples were sent to DNA Link Inc. (Seoul, South Korea) for DNA collection, extraction, and further analysis.

### Whole-exome sequencing

WES was performed on a Novaseq6000 using SureSelectXT Human All Exon V5. DNA quality was confirmed using 1% agarose gel electrophoresis and PicoGreen® dsDNA Assay (Invitrogen). The SureSelect sequencing library was prepared by complying with the manufacturer’s instructions (Agilent SureSelectXT Human All Exon V5). Genomic DNA (200 ng) in 50 μl EB buffer was fragmented to 150 bp size using a Covaris-S2 instrument (Covaris). Sequencing adapters were ligated to the DNA fragment according to the manufacturer’s protocol (Agilent). The adapter-ligated DNA was amplified by PCR. A hybridisation buffer was prepared by mixing SureSelect hyb #1, #2, #3, and #4 reagents (Agilent).

The amplified DNA fragment was concentrated to 750 ng in 3.4 μl, and SureSelect blocks #1, #2, and #3 reagents (Agilent) were added. The DNA blocking agent mixture and hybridisation buffer were incubated at 95 °C for 5 min and then at 65 °C for 10 min. An RNase block (Agilent) was added to the SureSelect oligo capture library (Agilent) and incubated at 65 °C for 2 min. After the hybridisation buffer was added, a DNA blocking agent mix was added to the capture library, and the mixture was incubated at 65 °C for 24 h in a thermal cycler. Streptavidin-coated beads (50 μl), Dynabeads MyOne Streptavidin T1 (Invitrogen), were washed three times with 200 ml of SureSelect binding buffer (Agilent) and resuspended in 200 ml of binding buffer. The hybridisation mixture was added to the bead suspension and incubated for 30 min at room temperature while mixing. Beads were washed with 500 ml of SureSelect wash buffers #1 and #2 (Agilent), and DNA was eluted with 30 µl of nuclease-free water. The captured library was amplified to add index tags.

After QPCR was performed using the SYBR Green PCR Master Mix (Applied Biosystems, Thermo Fischer Scientific), the libraries tagged with equimolar amounts in the pool were combined. The Illumina Novaseq 6000 system was used according to the protocol provided for 2×100 sequencing.

### WES data segregation analysis and variant filtering

Variants were identified from the WES results. Only variants in protein-coding transcripts were considered. Pedigree analysis of the family alluded to two possible inheritance patterns: autosomal dominant and X-linked dominant. To identify variants corresponding to those inheritance scenarios, we searched for heterozygous variants in the affected family members, but absent in unaffected members. After segregation analysis, we subsequently collected variants with minor allele frequency (MAF) < 5% in the Asian genome or have no reported MAF. Next, we applied additional filters to exclude the variants annotated as LOW & MODIFIER impact in SnpEff [27] (Supplement Table 1). The remaining 4 variants were subjected to in silico mutation impact analysis.

### In silico mutation impact analysis

To identify deleterious mutations that may cause pathogenicity of hypoplastic AI, we used Sorting Intolerant From Tolerant (SIFT) [28] and Polymorphism Phenotyping v2 (PolyPhen2) [29]. For the 4 missense variants, all possible non-redundant protein sequences in Ensembl GRCh37.p13 were analysed. These methods help assess mutational impacts through evolutionary analysis of protein sequences. Specifically, SIFT uses sequence conservation across multiple species with the assumption that a mutation in a highly conserved site is intolerable. PolyPhen2 utilises sequence co-evolution and protein structure to predict mutational impact. The SIFT and PolyPhen2 scores for variants of the 4 candidate genes were obtained using SnpEff [27], which uses precalculated scores in DbNsfp [30]. The candidate genes in which variants were specified with mutation impact analysis are summarised in Table 1.

**Table 1:** Mutation impact analysis results for candidate genes; SIFT and PolyPhen2 results of candidate variants on canonical transcripts

Gene	UniProt ID	Transcript ID	Protein change	SIFT	PolyPhen2
*MROH7*	Q68CQ1	ENST00000421030	A1313P	Damaging	Possibly damaging
*FAT2*	Q9NYQ8	ENST00000261800	R3318Q	Tolerated	Benign
*MTERF2*	Q49AM1	ENST00000240050	A31G	Damaging	Benign
*HCCS*	P53701	ENST00000321143	V64M	Damaging	Probably damaging

### In silico analysis of mutation impact on protein structure

To study the effect of mutations in Val64Met on the protein structure of HCCS, we employed the 3D structure of HCCS predicted by AlphaFold2, a state-of-the-art approach for predicting protein structure [31]. We found the interacting residues of Val64 using Protein Contacts Atlas, a tool for studying protein structures at atomic resolution utilising residue-residue interaction networks [32]. The relative solvent accessibility of the residues was quantified using Naccess calculating the atomic accessible surface established by rolling a solvent molecule over the van der Waals surface [33].

### Large population-based mutation impact analysis

To measure the harmful effect of abnormal function of the candidate genes, we used loss-of-function (LoF) observed/expected upper bound fraction [34] and the probability of being LoF intolerant (pLI) [35] by employing the genome aggregation database (gnomAD) v2.1.1 dataset [34], which spans 125,748 exome sequences and 15,708 whole-genome sequences from unrelated individuals sequenced as part of various disease-specific and population genetic studies. In addition, the missense *z*-score, which measures negative selection on missense mutation [36] of candidate genes, was also collected from the precalculated score in gnomAD v2.1.1 dataset.

### Literature-based association analysis of candidate genes

To query publication articles on specific genes and diseases, we used PubTator Central [37] and queried papers with Entrez ID of each candidate gene. Next, we queried “MESH:D00567”, which is the MESH term of AI. Lastly, we queried papers on both candidate gene ID and “MESH:D00567”. To measure the significance of co-citation rate between candidate genes and AI, we used Fisher’s exact test and considered *p* < 0.05 as significant.

### Function relationship analysis of HCCS

DAVID v6.8 [38] was used to perform GO term enrichment analysis for *HCCS*. To analyse the functional terms and pathways associated with the variants, we expanded the list of genes based on functional association using STRING v11.0 [39]. We extracted the first neighbour genes of *HCCS* in the STRING network with a 500 confidence level. Forty neighbour genes were identified (Supplement Table 2). The following categories were used in DAVID analysis: for “Gene_Ontology”: “GOTERM_BP_DIRECT”, “GOTERM_MF_DIRECT”, and “GOTERM_CC_DIRECT”; for “pathway”: “KEGG”, “REACTOME”. Table 2 presents functions significantly enriched in *HCCS* at *p* < 0.05.

**Table 2:** Function enrichment analysis result of HCCS and its STRING neighbouring genes

Category	Term	Count	%	Genes	*p*-value
GOTERM_CC_DIRECT	GO:0,005,743 ~ mitochondrial inner membrane	14	34.15	*TIMM8A*, *MTCH2*, *MRPS26*, *TIMMDC1*, *FECH*, *COX15*, *TIMM23*, *HCCS*, *MRPL35*, *CPT2*, *SCO1*, *CYCS*, *CYC1*, *COX10*	3.75 × 10^−12^
GOTERM_CC_DIRECT	GO:0,005,739 ~ mitochondrion	18	43.9	*TIMM8A*, *MTCH2*, *MRPS26*, *TIMMDC1*, *FECH*, *COX15*, *TIMM23*, *HCCS*, *GFER*, *MRPL35*, *CLYBL*, *CHCHD4*, *MSRA*, *CPT2*, *SCO1*, *CYCS*, *CYC1*, *COX10*	4.71 × 10^−10^
KEGG_PATHWAY	hsa00860: porphyrin and chlorophyll metabolism	6	14.63	*FECH*, *COX15*, *HMOX1*, *HCCS*, *COX10*, *HMOX2*	1.91 × 10^−8^
GOTERM_CC_DIRECT	GO:0,005,758 ~ mitochondrial intermembrane space	5	12.2	*CHCHD4*, *TIMM8A*, *TIMM23*, *CYCS*, *GFER*	1.85 × 10^−5^
REACTOME_PATHWAY	R-HSA-1268020: mitochondrial protein import	5	12.2	*CHCHD4*, *TIMM8A*, *TIMM23*, *CYC1*, *GFER*	2.06 × 10^−5^
GOTERM_BP_DIRECT	GO:0,008,535 ~ respiratory chain complex IV assembly	3	7.32	*SCO1*, *COX15*, *COX10*	2.22 × 10^−4^
GOTERM_MF_DIRECT	GO:0,005,507 ~ copper ion binding	4	9.76	*MOXD1*, *CCS*, *SCO1*, *ALB*	2.69 × 10^−4^
GOTERM_BP_DIRECT	GO:0,055,114 ~ oxidation–reduction process	8	19.51	*CHCHD4*, *MSRA*, *MOXD1*, *CCS*, *COX15*, *CYCS*, *HCCS*, *GFER*	3.18 × 10^−4^
REACTOME_PATHWAY	R-HSA-189451: Heme biosynthesis	3	7.32	*FECH*, *COX15*, *COX10*	4.96 × 10^−4^
GOTERM_BP_DIRECT	GO:0,006,123 ~ mitochondrial electron transport, cytochrome c to oxygen	3	7.32	*COX15*, *CYCS*, *COX10*	9.23 × 10^−4^
GOTERM_BP_DIRECT	GO:0,006,783 ~ heme biosynthetic process	3	7.32	*FECH*, *COX15*, *COX10*	1.02 × 10^−3^
GOTERM_BP_DIRECT	GO:0,045,333 ~ cellular respiration	3	7.32	*COX15*, *CYCS*, *COX10*	1.02 × 10^−3^
GOTERM_MF_DIRECT	GO:0,015,035 ~ protein disulphide oxidoreductase activity	3	7.32	*CHCHD4*, *CCS*, *GFER*	1.32 × 10^−3^
GOTERM_MF_DIRECT	GO:0,020,037 ~ heme binding	4	9.76	*HMOX1*, *CYCS*, *CYC1*, *HMOX2*	3.58 × 10^−3^
GOTERM_MF_DIRECT	GO:0,004,392 ~ heme oxygenase (decyclizing) activity	2	4.88	*HMOX1*, *HMOX2*	4.50 × 10^−3^
GOTERM_MF_DIRECT	GO:0,045,155 ~ electron transporter, transferring electrons from CoQH2-cytochrome c reductase complex and cytochrome c oxidase complex activity	2	4.88	*CYCS*, *CYC1*	4.50 × 10^−3^
GOTERM_BP_DIRECT	GO:0,006,788 ~ heme oxidation	2	4.88	*HMOX1*, *HMOX2*	4.52 × 10^−3^
GOTERM_BP_DIRECT	GO:0,006,784 ~ heme a biosynthetic process	2	4.88	*COX15*, *COX10*	4.52 × 10^−3^
GOTERM_CC_DIRECT	GO:0,070,069 ~ cytochrome complex	2	4.88	*COX15*, *COX10*	6.41 × 10^−3^
GOTERM_BP_DIRECT	GO:0,042,167 ~ heme catabolic process	2	4.88	*HMOX1*, *HMOX2*	1.57 × 10^−2^
GOTERM_MF_DIRECT	GO:0,015,450 ~ P-P-bond-hydrolysis-driven protein transmembrane transporter activity	2	4.88	*TIMM23B*, *TIMM23*	1.79 × 10^−2^
REACTOME_PATHWAY	R-HSA-189483: Heme degradation	2	4.88	*HMOX1*, *HMOX2*	1.84 × 10^−2^
GOTERM_CC_DIRECT	GO:0,005,744 ~ mitochondrial inner membrane presequence translocase complex	2	4.88	*TIMM23B*, *TIMM23*	1.91 × 10^−2^
GOTERM_BP_DIRECT	GO:0,006,979 ~ response to oxidative stress	3	7.32	*MSRA*, *HMOX1*, *HMOX2*	2.56 × 10^−2^
GOTERM_MF_DIRECT	GO:0,015,266 ~ protein channel activity	2	4.88	*TIMM23B*, *TIMM23*	2.67 × 10^−2^
REACTOME_PATHWAY	R-HSA-611105: respiratory electron transport	3	7.32	*SCO1*, *CYCS*, *CYC1*	2.80 × 10^−2^
KEGG_PATHWAY	hsa00190: oxidative phosphorylation	3	7.32	*COX15*, *CYC1*, *COX10*	3.30 × 10^−2^
GOTERM_BP_DIRECT	GO:0,006,122 ~ mitochondrial electron transport, ubiquinol to cytochrome c	2	4.88	*CYCS*, *CYC1*	3.34 × 10^−2^
GOTERM_BP_DIRECT	GO:0,030,150 ~ protein import into mitochondrial matrix	2	4.88	*TIMM23B*, *TIMM23*	3.78 × 10^−2^
GOTERM_CC_DIRECT	GO:0,031,305 ~ integral component of mitochondrial inner membrane	2	4.88	*TIMM23B*, *TIMM23*	4.20 × 10^−2^
GOTERM_CC_DIRECT	GO:0,070,469 ~ respiratory chain	2	4.88	*CYCS*, *CYC1*	4.20 × 10^−2^
REACTOME_PATHWAY	R-HSA-917937: iron uptake and transport	2	4.88	*HMOX1*, *HMOX2*	4.53 × 10^−2^

## Results

### Patient characteristics

**Fig. 1:**
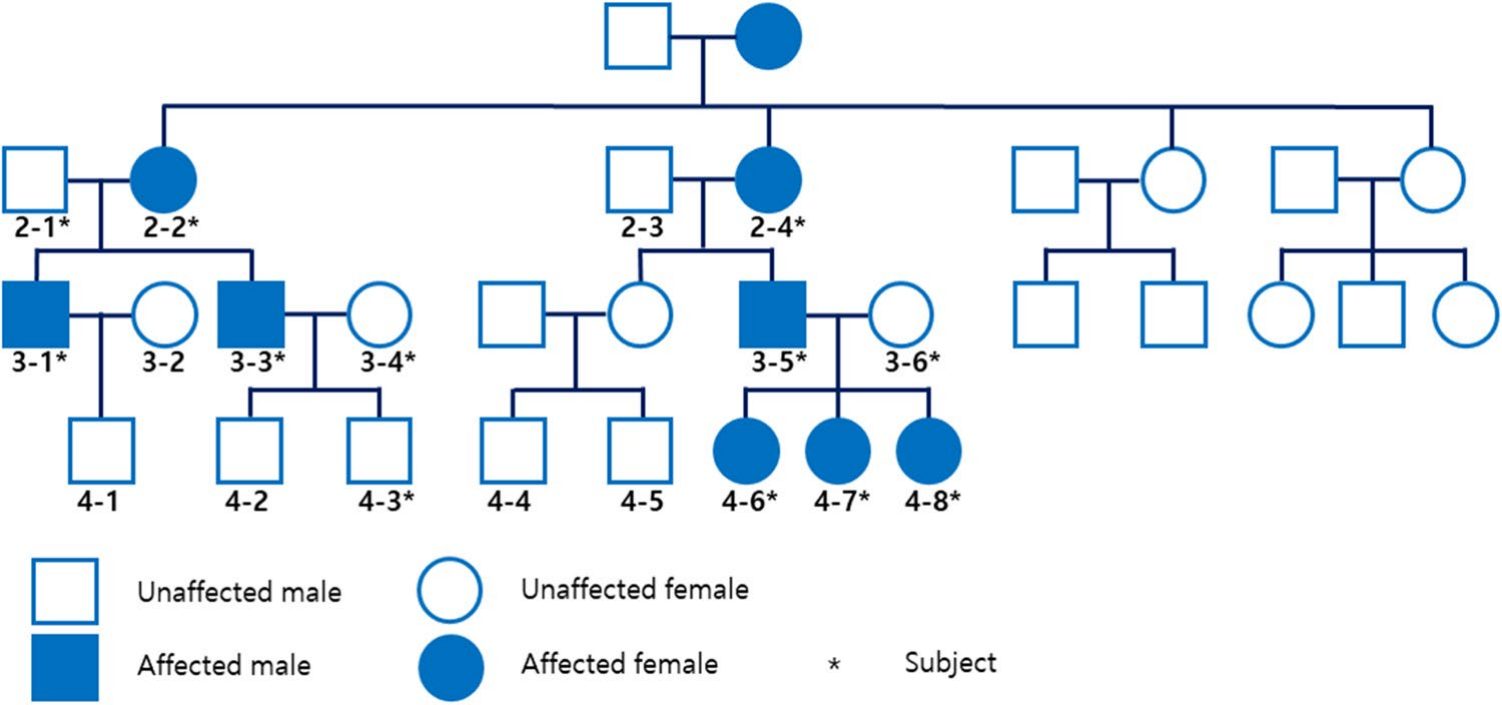
Pedigree of the family of patients with amelogenesis imperfecta (AI). Inherited AI has been in the family for over four generations. The genetic penetration pattern of this family showed the possibility of autosomal dominance and X-linked traits

A total of 12 samples were used for WES analysis, consisting of samples from 7 females and 5 males, 8 of which were AI patients. All patients had permanent dentition, except for a 9-year-old patient (Fig. 1; individual 4-8) with mixed dentition.

### Clinical manifestation

**Fig. 2:**
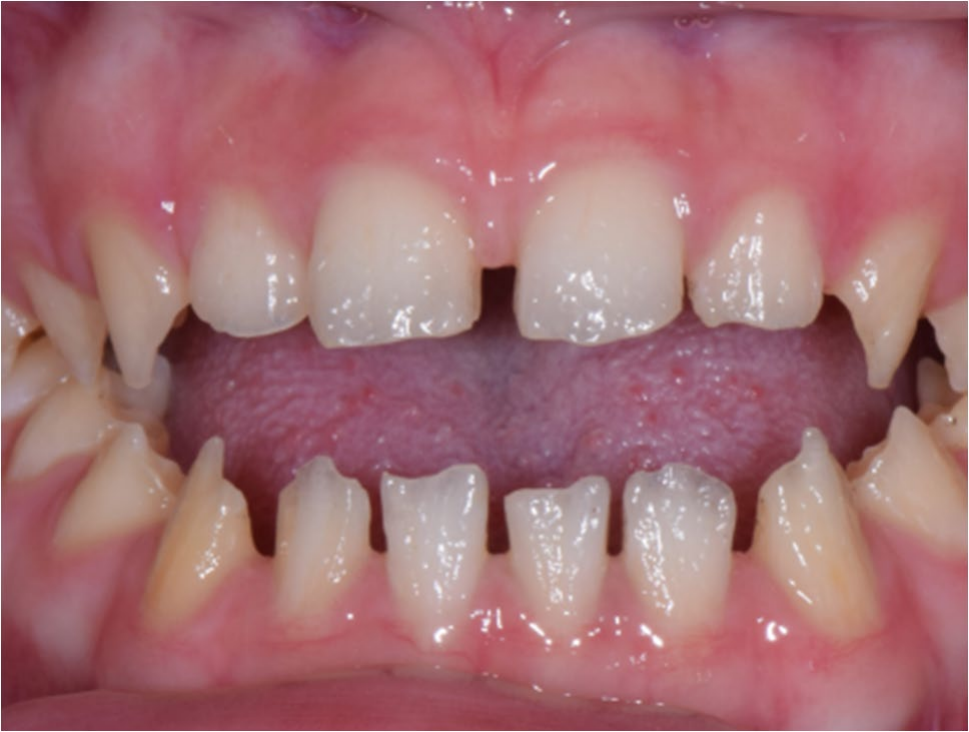
Clinical picture of the individual (4–7) in the family. The enamel was thin or missing, and the dentin appeared to be exposed. The overall shape was abnormal, and the surface was rough. Because of the lack of enamel thickness, the mesiodistal width of the teeth was smaller than normal; hence, there was no contact between adjacent teeth. These characteristics correspond to the hypoplastic type amelogenesis imperfecta (AI)

**Fig. 3:**
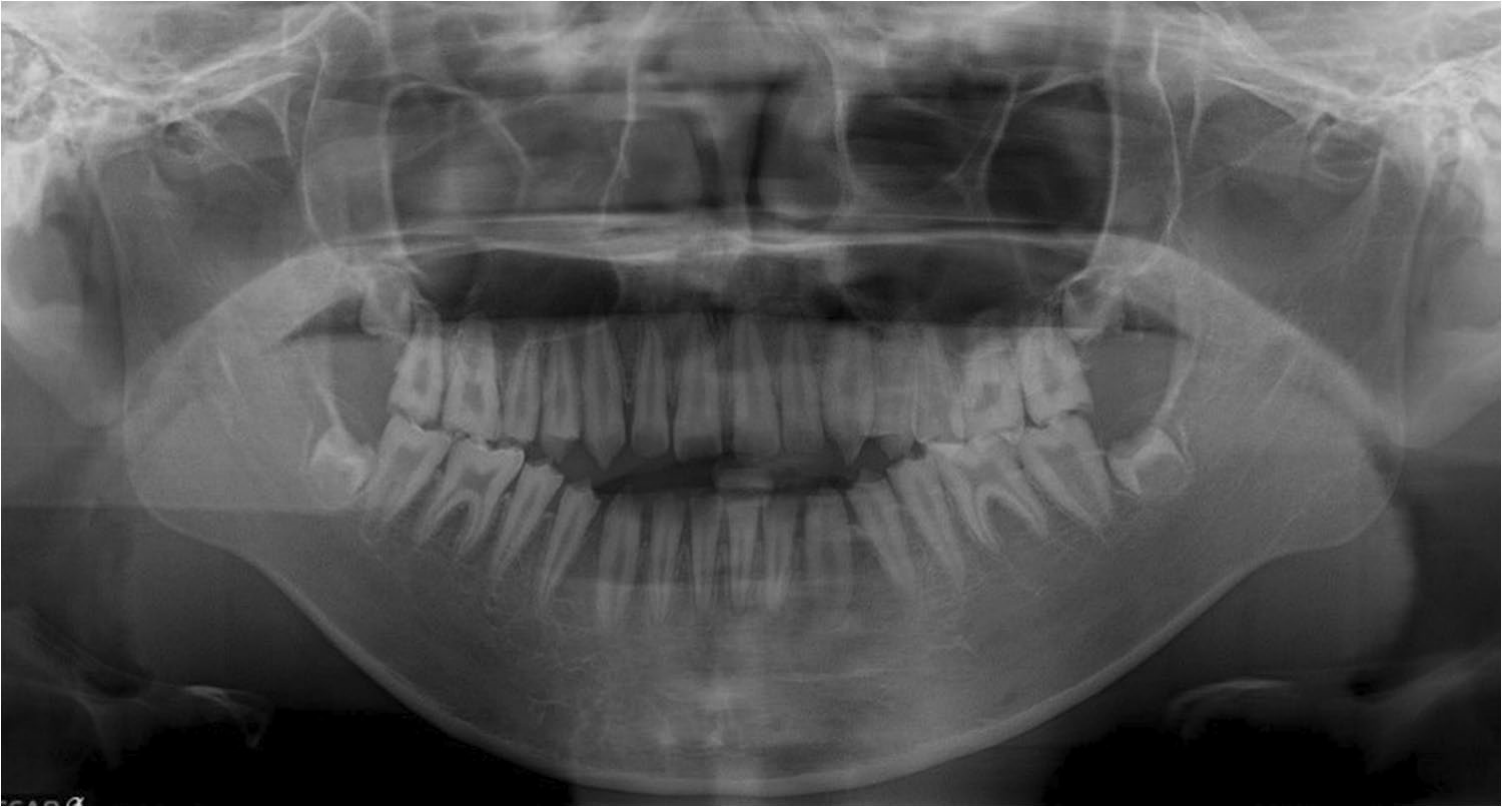
Panoramic radiograph of the individual (4–7) in the family. In the panorama radiograph, enamel was hardly observed, and a very thin layer of enamel was observed only in some teeth. A space between the teeth was also observed because of the lack of enamel formation

According to clinical and radiographic assessment, all the patients showed hypoplastic type AI in which all parts of the enamel seem not to reach normal thickness but were hard and translucent in radiographs. Clinically, the enamel was thin, and the mesiodistal width of the tooth was small such that the adjacent teeth were not in contact with each other (Fig. 2). The dentin showed normal features in radiographs, and the enamel was either very thin or not observed (Fig. 3). There was no enamel layer in severe cases, and there was severe wear on all posterior teeth in individuals 3-3 and 3-5 from the family. The overall shapes were not normal, and the surface was rough. Unusual pits and grooves were found on the occlusal surface.

### Candidate genes discovered in WES analysis

WES was performed on all the consenting individuals of the family. The AI patients from the family showed two possible inheritance patterns: autosomal dominant and X-linked dominant. A total of 20 variants were identified through segregation analysis: 14 variants were accounted for by autosomal dominant inheritance, and 6 variants are explained by X-linked dominant inheritance (Table 3 and Supplement Table 3). Four of the 20 variants met all the filtering criteria (Table 1). Three missense variants in these three genes are rare in the Asian population: rs143029488 in *MROH7* with Asian MAF of 0.0105, rs7718054 in *FAT2* with Asian MAF of 0.0437, and rs191165757 in *HCCS* with Asian MAF of 0.0017. A missense variant (ENST00000240050; c.92C>G) in *MTERF2* had an undefined MAF (Table 3). For example, the variant in *HCCS* showed a heterozygous genotype in the affected family member 2-2, but was not present in the unaffected family member 3-6 (Table 4). All 4 variants were used in the subsequent mutation impact analysis.

**Table 3:** List of variants after filtration. *dbSNP_id*, variant id in dbSNP; *CHR*, chromosome; *POS*, base pair position; *REF*, reference allele; *ALT*, alternative allele; *Transcript ID*, canonical transcript ID; *ASN MAF*, alternative allele frequency in 1000Gp1 Asian descendent samples; *MAF*, alternative allele frequency in whole 1000Gp1 data

Gene	dbSNP_id	CHR	POS	REF	ALT	Transcript ID	Effect	ASN MAF	MAF
*MROH7*	rs143029488	1	55,175,825	G	C	ENST00000421030	Missense variant	0.0105	0.0027
							p.Ala1313Pro		
*FAT2*	rs7718054	5	150,908,812	C	T	ENST00000261800	Missense variant	0.0437	0.0691
							p.Arg3318Gln		
*MTERF2*	No_id	12	107,372,401	G	C	ENST00000240050	Missense variant	Not_defined	Not_defined
							p.Ala31Gly		
*HCCS*	rs191165757	X	11,133,044	G	A	ENST00000321143	Missense variant	0.0017	0.0006
							p.Val64Met

**Table 4:** Genotypes of 4 variants from segregation analysis in 12 family members; minor or risk allele in segregation analysis

Family member	Sex	AI phenotype	MROH7 (C)^1^	FAT2 (T)^1^	MTERF2 (C)^1^	HCCS (A)^1^
2–1	Male	Unaffected	G/G	C/C	G/G	G/-
2–2	Female	Affected	G/C	C/T	G/C	G/A
2–4	Female	Affected	G/C	C/T	G/C	G/A
3–1	Male	Affected	G/C	C/T	G/C	A/-
3–3	Male	Affected	G/C	C/T	G/C	A/-
3–4	Female	Unaffected	G/G	C/C	G/G	G/G
3–5	Male	Affected	G/C	C/T	G/C	A/-
3–6	Female	Unaffected	G/G	C/C	G/G	G/G
4–3	Male	Unaffected	G/G	C/C	G/G	G/-
4–6	Female	Affected	G/C	C/T	G/C	G/A
4–7	Female	Affected	G/C	C/T	G/C	G/A
4–8	Female	Affected	G/C	C/T	G/C	G/A

### In silico mutation impact analysis of the variants

**Fig. 4:**
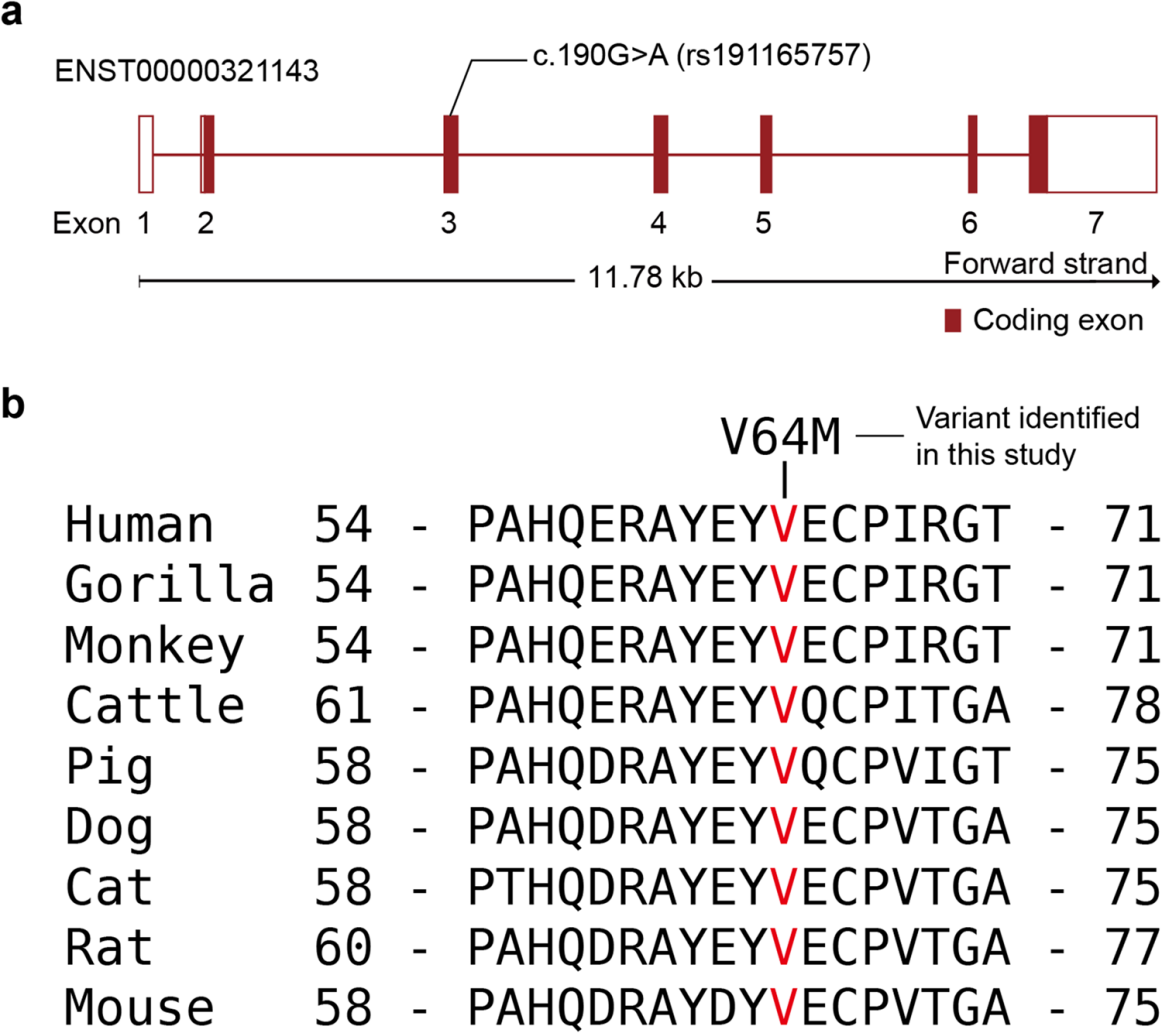
Schematic view of structure of *HCCS*, and multiple-sequence alignment result of *HCCS*. **a** Coding exons are shown as red boxes and introns are shown as red lines. The missense variant (c.190G > A, p.Val64Met, rs191165757) identified in this study is shown above the exons. **b** Amino acid sequence alignment of *HCCS* on various mammal species. The mutated Val64 residue is indicated with red characters

For the 4 genes (*MROH7*, *MTERF2*, *FAT2*, and *HCCS*) with filtered variants, we conducted mutation impact analysis using two distinct computational tools: SIFT [28] and PolyPhen2 [29]. For the 4 missense variants, canonical Ensembl sequences were analysed (Table 1). A missense variant in *MROH7* was predicted to be damaging by SIFT and possibly damaging by PolyPhen2 (Table 1). A variant in *FAT2* was predicted to be benign by both tools. The variant in *MTERF2* was predicted to have deleterious effects by SIFT but not by polyphen2. A missense variant (c.190G>A, p.Val64Met) in HCCS was predicted to be confidently deleterious by both tools. It implied that the variant is in evolutionarily conserved position and might be under strong selective pressure. In Fig. 4a, for example, the missense variant is located in exon 3 of *HCCS,* and valine at amino acid position 64 in this exon is well-conserved in mammal orthologs (Fig. 4).

Next, to measure the putative impact of abnormal function of each gene, we conducted large population-based mutation impact analysis of the 4 candidate genes. From 125,748 exome sequences and 15,708 whole-genome sequences in the genome aggregation database [34], we measured organism-level impact of each candidate gene’s inactivation using LoF observed/expected upper bound fraction [34] and the pLI [35] (Table 5). Loss-of-function observed/expected upper bound fraction (LOEUF) and pLI measure the degree of selection against predicted LoF (pLoF) variation. Low LOEUF scores and high pLI scores imply strong selection against pLoF variation in a given gene, which means the malfunction of the gene has great impact on patient survival. We found that *HCCS* had the most deleterious effect when its function was lost among the 4 candidate genes. *HCCS* had the lowest LOEUF value and the highest pLI value (0.41 and 0.89, respectively). In addition, measurement of the impact of missense mutation showed that *HCCS* had the highest intolerance (missense *z*-score [36] = 1.2). For further validation of the deleterious effect of abnormal gene function, we next checked the number of reported pathogenic variants in each candidate gene using ClinVar [40], which is a public archive of reports of the relationships among human variations and phenotypes, with supporting evidence. Among the 4 candidate genes, only *HCCS* had reported pathogenic variants that are related to microphthalmia with linear skin defect syndrome (MLS), which is reported to occasionally have abnormal dental enamel morphology as a symptom [41]. Therefore, we conducted further evaluation of *HCCS* using in silico analysis of protein structure.

**Table 5:** Large population based-mutation impact analysis results for candidate genes; “ClinVar variants” is the number of reported ClinVar pathogenic and likely pathogenic variants

Gene	LOEUF	pLI	Missense *z*-score	ClinVar variants
*MROH7*	0.98	0	− 0.08	0
*FAT2*	0.51	0	0.72	0
*MTERF2*	1.31	0	− 0.24	0
*HCCS*	0.41	0.89	1.2	4

### Potential effect of HCCS mutation on protein 3D structure

**Fig. 5:**
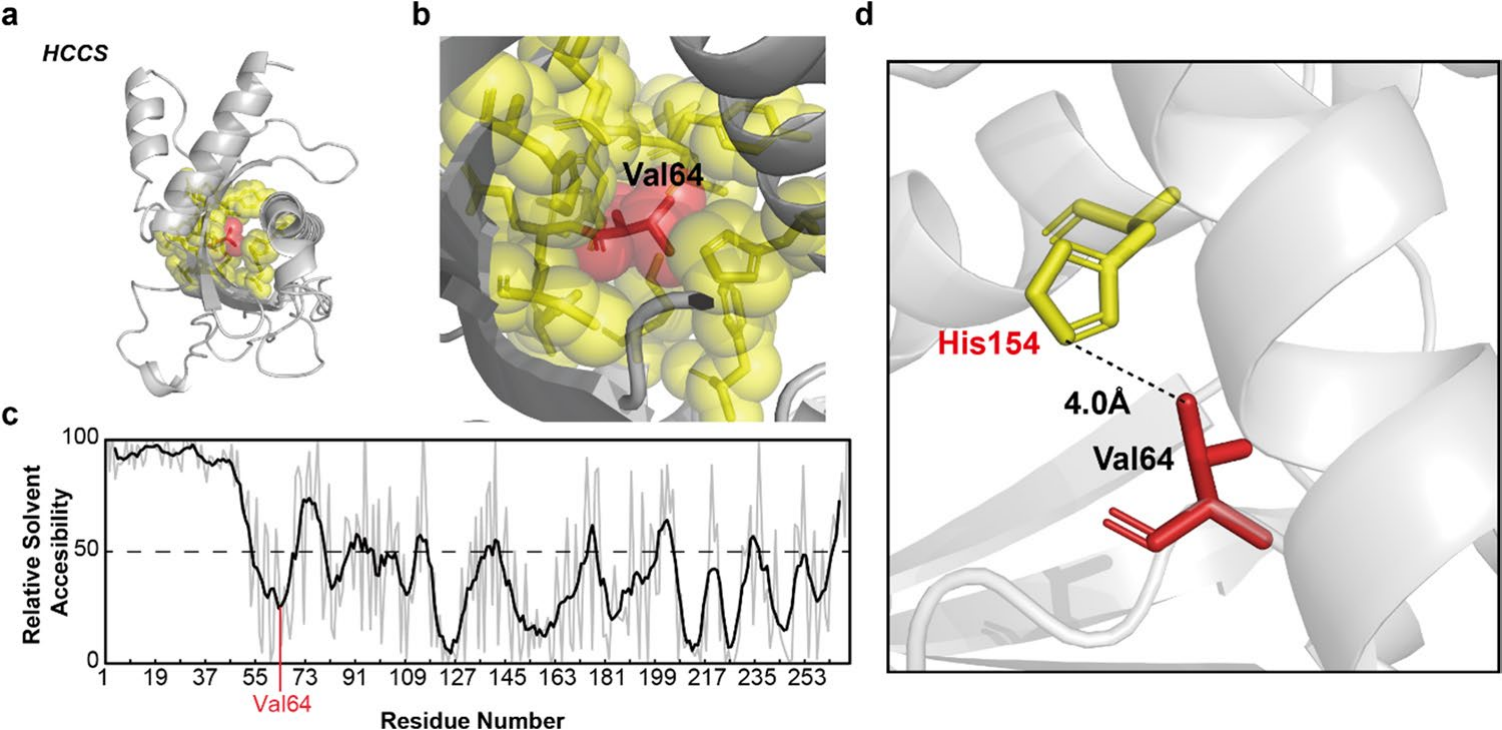
In silico analysis of the variant (Val64Met)’s effect on *HCCS* protein. **a** Three-dimensional structure of HCCS generated with the PyMOL program. The red bubbles are valine 64 (Val64) and the yellow bubbles are 7 interacting residues of Val64. **b** The Val64 residue is packed in 7 amino acid residues. **c** Relative solvent accessibility plot of residues in *HCCS*. **d** The histidine 154 (His154) residue interacting with sidechains of Val64

The Val64Met mutation is predicted to have a negative impact on protein structure. In silico analysis of the tertiary structure of the HCCS revealed that the Val64 residue is located in a highly packing region in the 3D structure of *HCCS* (Fig. 5a, red area). The Val64 has direct interactions with seven amino acids (Fig. 5a, b, yellow region). In addition, Val64 residue is located in a deeply buried region and has 8.00 relative solvent accessibility (RSA, Fig. 5a, c). A residue with a low RSA value tends to have a small exposure to solvent and is usually located in an inner space of a protein. The Val64Met substitution, a change to a larger side chain in a low RSA position, could disrupt the protein folding. Furthermore, His154, one of the direct interacting residues of Val64, is known as a critical region for the catalytic activity of *HCCS* [42]. Therefore, the Val64Met substitution can affect interaction with the His154 and potentially impair the protein function.

### Evaluation of the effect of genes thorough literature-based association analysis

To investigate whether there is evidence on the relationship between candidate genes and AI, we used a PubMed text-mining method to conduct an independent literature-based association analysis of the 4 candidate genes. We found that only *HCCS* was significantly co-cited with AI in PubMed articles (*p*-value = $3.23\times {10}^{-10}$, Table 6). *HCCS* was co-mentioned with AI in 6 articles among 906 articles queried for AI. To test whether this number is higher than expected, we measured the enrichment odds ratio and *p*-values between the articles mentioning HCCS and the articles mentioning AI using Fisher’s exact test.

**Table 6:** Literature-based association analysis results for candidate genes. OR is the odds ratio between the articles mentioning the query gene and 906 articles mentioning amelogenesis imperfecta (AI). Enrichment p-value was calculated using Fisher’s exact test in the articles mentioning the query gene and 906 articles mentioning AI

Gene	Articles queried by the gene	AI and gene co-mentioned articles	OR	Enrichment *p*-value
*MROH7*	22	0	0	1
*FAT2*	304	0	0	1
*MTERF2*	56	0	0	1
*HCCS*	2349	6	76.06	3.23E-10

### Function enrichment analysis of HCCS and its neighbour genes

To understand candidate genes’ functional roles in the amelogenesis process, we analysed the functional terms of *HCCS* and its neighbour genes in the STRING network. The analysis yielded three notable categories: GO, KEGG, and REACTOME. We discovered 32 enriched functional terms for *HCCS* (Table 2). The topmost overrepresented term of *HCCS* was “mitochondrial inner membrane”.

## Discussion

This study is the first to combine WES with bioinformatic tools to report hypoplastic AI in a Korean family. A missense mutation (rs191165757) in *HCCS* was identified through segregation analysis of WES data and variant filtering. Interestingly, we found that the MAF for the HCCS variant (rs191165757, MAF = 0.0017) was similar to the AI incidence rate (from 1:714 to 1:14,000 [2]). The similarity between the MAF for the *HCCS* variant and the AI incidence rate as well as the inheritance pattern of *HCCS* is intriguing in that this mutation may trigger AI with only a single occurrence.

Until now, most of the variants found in AI were autosomal dominant and autosomal recessive, and the only X-linked inherited mutation was that of *AMELX*. *AMELX* is an amelogenin gene, and its defect causes enamel hypoplasia. *AMELX* is involved in both normal thickness enamel formation and normal mineralisation processes [9]. *HCCS*, a variant of which was found in this study, is a newly discovered gene by X chromosome analysis. Although *HCCS* is close to genomic region of known AI-related genes, *AMELX*, no harmful variant was detected in *AMELX* from affected family members. Therefore, we suppose that dysfunction of *HCCS* itself might be related to critical defects in dental morphology. During amelogenesis, the number of ameloblasts is one of the factors that affect the amount of enamel produced. In the transition and maturation stages of amelogenesis, 25% of ameloblasts undergo apoptosis [16]. Hobson noted that HCCS protein is involved in regulating apoptosis and cell necrosis. Problems in the apoptosis process due to *HCCS* mutation could be a factor contributing to enamel defect in patients [43].

Additional evidence of the potential pathogenicity of abnormal function of *HCCS* has been provided in a large-scale population study. We found that loss of function in HCCS had a low LOEUF score (0.41, Table 5), which means it has a fatal effect on affected individuals. This corresponds to the male lethality of MLS syndrome, which is caused by loss of function in *HCCS* [41]. Although the discovered variant is not protein-truncating, the amino acid sequence changed, and the variant was consistently expected to have deleterious effect through in silico mutation impact analysis (Table 1). Therefore, we suppose that the missense variant in *HCCS* might be related to critical defects in dental morphology.

Through in silico mutation impact analysis based on sequence conservation, the missense mutation in *HCCS* was predicted to be deleterious due to Val64 which is highly conserved across the species. This is supported by position of Val64 residue in 3D protein structure of *HCCS* predicted by AlphaFold. We observed that the mutated residue (Val64) in HCCS is predicted to have direct interaction with His154 residue. In the mitochondria, HCCS catalyse cytochrome c and heme to matured holocytochrome c, and the conserved histidine (His154) in HCCS provides the key ligand to the heme iron [42]. Therefore, it is postulated that Val64Met substitution in *HCCS* negatively affects the synthesis of holocytochrome c, an essential mitochondrial electron carrier and an important component of the apoptosis pathway, leading to adverse effect on the mitochondria function. It suggests that one of the unknown pathogenic mechanisms of AI is underlying mitochondria dysfunction.

By discovering defective mitochondrial genes in AI patients, this study advances efforts to elucidate the role of mitochondria in amelogenesis. We found that *HCCS* and its first neighbour genes in a protein-protein network were significantly enriched in functions and pathways related to mitochondria: “mitochondrial protein import pathway”, “respiratory chain complex IV assembly”, and “mitochondrial electron transport” (Table 2). There are several evidences of the functional relationship between mitochondria and amelogenesis. At the maturation stage of amelogenesis, the transport of Ca^2+^ and PO_4_^3−^ is increased, and the width and thickness of the enamel crystal are expanded [44]. In this process, ameloblasts alternate between a ruffle-ended (RE) border and a smooth-ended (SE) border [19]. In some reports, the number of mitochondria in ameloblasts increased from the secretory stage to the maturation stage. In addition, mitochondria themselves also increase in size during maturation and have an efficiency that provides a lot of energy to cells [45]. Cytochrome oxidase (CO) involved in oxidative phosphorylation reflects the functional activity of mitochondria. The proportion of CO-reactive mitochondria is significantly higher in SE ameloblasts than in RE ameloblasts. This indicates that a higher energy level is required to convert from SE to RE or that SE ameloblasts may still play an unknown, highly energy-demanding role [46]. Costiniti reported an increase in oxidative phosphorylation, a measure that quantifies the oxygen consumption rate of mitochondria at the maturation stage, indicating an increase in energy demand [47]. Enamel mineralisation depends on the transport system of Ca^2+^ and PO_4_^3−^ and other ions by ion transport, channels, and pumps [19, 44, 48]. During the crystallisation of the enamel in the maturation stage, pH change adjustments occur together to buffer acidification due to proton release [49]. Besides, in maturation stage ameloblasts, mitochondria accumulate near the apical end of the RE border and show a different localisation from the secretory ameloblasts [47]. This mitochondrial accumulation can contribute to the movement of ions required for mineralisation [50].

Even though the new findings of this study are from a limited condition, the association between genetic variation and cellular function can provide worthy information for the further study of AI to understand the physiology of amelogenesis and pathogenesis of AI. In particular, this study could help identify genes that play a role in the maturation stage of amelogenesis. The discovery of *HCCS* revealed a genetic link to the role of mitochondria in the process of amelogenesis. In this context, this study could help uncover the mineralisation process of the maturation stage ameloblasts. Since AI has several different subtypes, it is worthy to investigate one specific family due to the idiopathic origin of the disease. For further studies with a large number of affected individuals from different families, it is important to perform proper subtype grouping after careful consideration of clinical and radiographical examinations.

## Supplementary Information

Below is the link to the electronic supplementary material.

Supplementary file1 (XLSX 17 KB)

Supplementary file2 (XLSX 10 KB)
